# Orai1, 2, 3 and STIM1 promote store-operated calcium entry in pulmonary arterial smooth muscle cells

**DOI:** 10.1038/cddiscovery.2017.74

**Published:** 2017-11-27

**Authors:** Jian Wang, Chuyi Xu, Qiuyu Zheng, Kai Yang, Ning Lai, Tao Wang, Haiyang Tang, Wenju Lu

**Affiliations:** 1 State Key Laboratory of Respiratory Diseases, Guangzhou Institute of Respiratory Disease, The First Affiliated Hospital of Guangzhou Medical University, Guangzhou 510180, China; 2 Division of Translational and Regenerative Medicine, Department of Medicine, The University of Arizona, Tucson, AZ 85721-0202, USA

## Abstract

Previous studies have demonstrated that besides the classic canonical transient receptor potential channel family, Orai family and stromal interaction molecule 1 (STIM1) might also be involved in the regulation of store-operated calcium channels (SOCCs). An increase in cytosolic free Ca^2+^ concentration promoted by store-operated Ca^2+^ entry (SOCE) in pulmonary arterial smooth muscle cells (PASMCs) is a major trigger for pulmonary vasoconstriction and proliferation and migration of PASMCs. In this study, our data revealed the following: (1) in both rat distal pulmonary arteries and PASMCs, chronic hypoxia exposure upregulated the expression of Orai1 and Orai2, without affecting Orai3 and STIM1; (2) either heterozygous knockout of HIF-1*α* in mice or knockdown of HIF-1*α* in PASMCs abolished the hypoxic upregulation of Orai2, but not Orai1, suggesting the hypoxic upregulation of Orai2 depends on HIF-1*α*; and (3) using small interference RNA knockdown strategies, Orai1, 2, 3 and STIM1 were all shown to mediate SOCE in hypoxic PASMCs. Together, these results suggested that the components of SOCCs, including Orai1, 2, 3 and STIM1, may lead to novel therapeutic targets for the treatment of chronic hypoxia-induced pulmonary hypertension.

## Introduction

According to consensus of the Fifth World Symposium of Pulmonary Hypertension held in Nice, France, in 2013, chronic hypoxia-induced pulmonary hypertension (CHPH) belongs to group 3 of pulmonary hypertension (PH). PH group 3 is due to lung diseases and/or hypoxia, including chronic obstructive pulmonary disease, sleep-disordered breathing, alveolar hypoventilation disorders, diffuse parenchymal lung diseases, chronic exposure to high altitude and developmental abnormalities.^1^

CHPH is characterized by excessive contraction, proliferation and migration of pulmonary arterial smooth muscle cells (PASMCs), which progressively leads to the thickening and remodeling of distal pulmonary arteries (PAs). The increase of intracellular free calcium concentration ([Ca^2+^]_i_) is a major trigger for pulmonary vasoconstriction and the proliferation and migration of PASMCs.^2^ Among the multiple pathways that can lead to increase in [Ca^2+^]_i_, the hypoxia-induced enhanced store-operated Ca^2+^ entry (SOCE) through store-operated calcium channels (SOCCs) largely accounts for the elevated [Ca^2+^]_i_ in PASMCs.^3,4^ SOCCs are mainly constituted by canonical transient receptor potential channel (TRPC) and calcium release-activated calcium modulator (also called Orai).^5–8^ Orai consists of three members, Orai1, Orai2 and Orai3. Recent studies have revealed that Orai1 might be involved in the constitution of SOCCs, or regulates the function of SOCCs.^6,7,9^ In mesenteric artery smooth muscle cells, the expression level of Orai members are upregulated in proliferating cells.^10^ Orai1 consists of four transmembrane domains. Under resting conditions, Orai1 exists either as a homodimer or homotetramer; while upon activation, it forms a hexamer and mediates Ca^2+^ release-activated Ca^2+^ current (*I*_CRAC_), a highly Ca^2+^-selective and nonvoltage-gated current.^11–15^ The activation kinetics of Orai1 are relatively slow and determined by the rate of Ca^2+^ depletion as well as the translocation rate of both STIM1 and Orai1 to endoplasmic reticulum (ER)/sarcoplasmic reticulum (SR)–plasma membrane (PM) junctions. The duration of Orai1 activation can be sustained with prolonged store depletion.^16^ Among the three Orai homologs, Orai1 contributes the most to mediate SOCE.^17^ Soon after Orai1 was discovered in 2006, Orai2 was reported as another component of SOCCs.^17–19^ Similar with Orai1, Orai2 and Orai3 are highly Ca^2+^ selective corresponding to the characteristic of CRAC (Ca^2+^ release-activated Ca^2+^) channels.^20,21^ Orai3 was only found in mammals, with a tissue distribution at least as wide as that of Orai1.^22,23^ Orai3 combines with Orai1 to form a hexameric CRAC channel, and at least one native Orai1 subunit is contained in the complex.^24^ Orai1 and Orai3 arrange as pentamer to form the arachidonic acid-regulated calcium (ARC) channels, whose characteristics are similar to CRAC channels, but are store-independent.^25^ However, whether the Orais contribute to hypoxia-induced enhancement of SOCE remains largely unknown.

Stromal interaction molecule 1 (STIM1), a single-pass transmembrane protein, has been well known to predominantly localize in the ER/SR membrane where it acts as a Ca^2+^ sensor and mediates SOCE.^26–28^ Global deletion of STIM1 in mice is lethal indicating that STIM1 is indispensable in organismal physiology of mammals.^29^ The homolog STIM2 shares 61% structural homology with STIM1.^30^ When Ca^2+^ depleted in ER/SR calcium pool, STIM1 departs ER/SR membrane and translocates to cell membrane, where it activates SOCCs and initials the SOCE.^5,26,31^ Previous studies have reported in HEK293, epithelial cells, SH-SY5Y nerve sarcoma cells or smooth muscle cells that silencing of *STIM1* gene could dominantly eliminate SOCE.^32,33^ Our previous study indicated that STIM1 was quantitatively more important than STIM2 in activation of SOCCs in distal PASMCs.^34^ Besides mediating SOCE, STIM1 also contributes to store-independent Ca^2+^ entry, more specifically the activation of arachidonic ARC-selective channels.^35,36^ ARC channels have very similar biophysical characteristics to SOCCs, have been shown to contribute to receptor-operated Ca^2+^ entry, and are also dependent on STIM1 for activation. However, ARC channels are dependent on a PM pool of STIM1, rather than ER/SR located STIM1.^36^ Moreover, unlike SOCCs, which consist of six homomeric Orai1 subunits, activated ARC channels consist of both Orai1 and Orai3 subunits. Recent investigations have revealed that STIM1 acts as a sensor of Ca^2+^ concentration in ER/SR and could also sense reactive oxygen species (ROS) overproduction, temperature variation, hypoxic stress and pH changes in the cells, indicating that STIM1 might be a stress sensor sensing a range of cellular stress condition.^37–40^ In our previous study, we elucidated that knockdown of STIM1 abolishes acute hypoxia (4% O_2_, 15 min)-induced enhancement of SOCE.^34^ Considering SOCE largely accounts for the elevated [Ca^2+^]_i_ in PASMCs, we hypothesized that STIM1 may also have an important role in prolonged hypoxia-induced SOCE.

Therefore, in this study, we further investigated the regulation and action of Orai family and STIM1 in chronic hypoxia-induced elevation of SOCE in PASMCs.

## Results

### Chronic hypoxia increased expression of Orai1 and Orai2, but not Orai3 and STIM1 in distal PA

**Figure 1:**
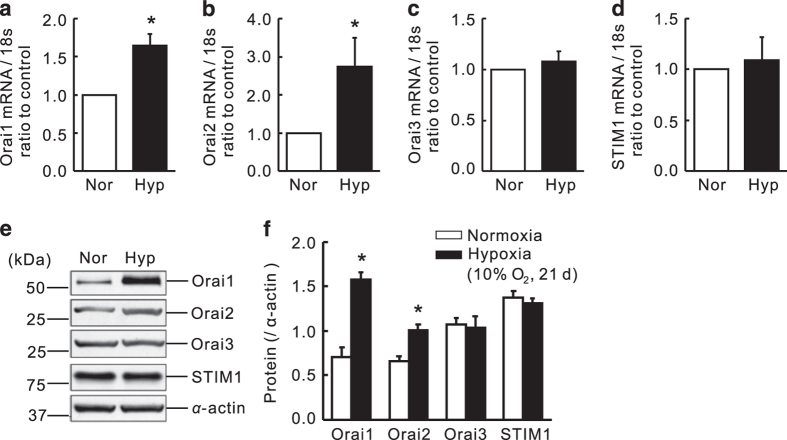
Expression of Orai and STIM1 in distal PAs from rats exposed to normoxia or hypoxia (10% O_2_) for 21 days. Orai1 (**a**), Orai2 (**b**), Orai3 (**c**) and STIM1 (**d**) mRNA relative to 18 s was measured by qRT-PCR. Orai1, 2, 3 and STIM1 proteins were determined by western blotting (**e** and **f**). Representative blots (**e**) and mean intensity (**f**) for Orai1, 2, 3 and STIM1 blots relative to *α*-actin. Bar values are mean±S.E.M. (*n*=5 in each group). *P<0.05 *versus* respective normoxic control. Brackets indicate ±S.E.

Distal PA were isolated from rats exposed to either normoxia or hypoxia (10% O_2_) for both mRNA and protein assessment. Results showed that hypoxia induced a 64.7±22.7% and 162.2±100.3% increase in Orai1 and Orai2 mRNA level, compared with those of their respective normoxic control, while not affecting the expression of Orai3 and STIM1 (Figures 1a–d). In protein level, chronic hypoxia led to a 125.9±62.1% and 51.1±11.5% increases in Orai1 and Orai2 expression, respectively, without affecting Orai3 and STIM1 protein expression (Figures 1e and f).

### Heterozygote of HIF-1*α* (*HIF-1*α*^+/−^*) transgenic mice exhibited abolished CH upregulation of Orai2, but not Orai1

**Figure 2:**
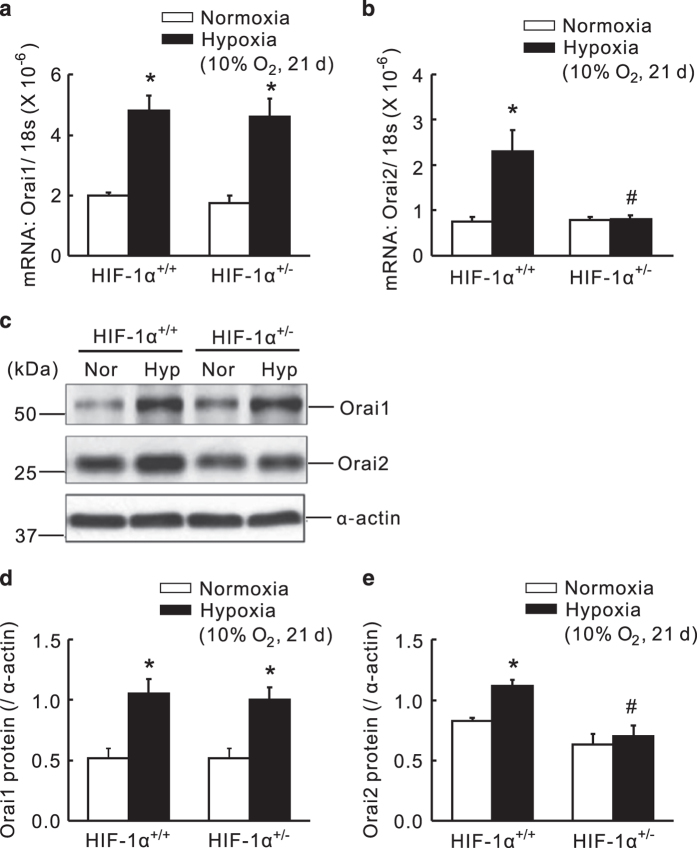
Knockdown of HIF-1*α* abolished the chronic hypoxia-upregulated Orai2 expression, while not affecting the upregulation of Orai1 in distal PAs of transgenic mice. *HIF-1*α*^+/+^* and *HIF-1*α*^+/−^* mice were subjected to chronic hypoxia (10% O_2_, 21 days) or normoxia. Orai1 (**a**) and Orai2 (**b**) mRNA relative to 18 s was measured by qRT-PCR. Orai1 and Orai2 proteins were determined by western blotting (**c**, **d** and **e**). Representative blots (**c**) and mean intensity (**d** and **e**) for Orai1 and Orai2 blots relative to *α*-actin. Bar values are mean±S.E.M. (*n*=5 in each group). *P<0.05 *versus* respective normoxic control. ^#^*P*<0.05 *versus* respective *HIF-1*α*^+/+^* control. Brackets indicate ±S.E.

Consider that HIF-1 acts as a master regulator in hypoxic PASMCs, we further determined whether CH upregulation of Orai1 and Orai2 is HIF-1*α*-dependent. As described before,^41^ HIF-1*α* heterozygous transgenic mice (*HIF-1*α*^+/−^*) and their littermate were subjected to either normoxic or chronic hypoxic exposure for establishment of CHPH mice model. First, in the distal PAs isolated from wild-type *HIF-1*α*^+/+^* mice, CH (10% O_2_, 21 days) increased Orai1 and Orai2 by 144.7±18.1% and 250.2±105.9% in mRNA levels, and 101.6±8.8% and 35.2±7.8% in protein levels, comparing with their respective normoxic controls (Figure 2). Then, in the distal PAs isolated from *HIF-1*α*^+/−^* mice, the expression of Orai2 on mRNA and protein levels were decreased by 64.5±9.9% and 38.5±11.9%, respectively, compared with those of *HIF-1*α*^*+/+*^*mice.

### Knockdown of HIF-1*α* by small interference RNA transfection abolished the hypoxic upregulation of Orai2, but not Orai1 in PASMCs

**Figure 3:**
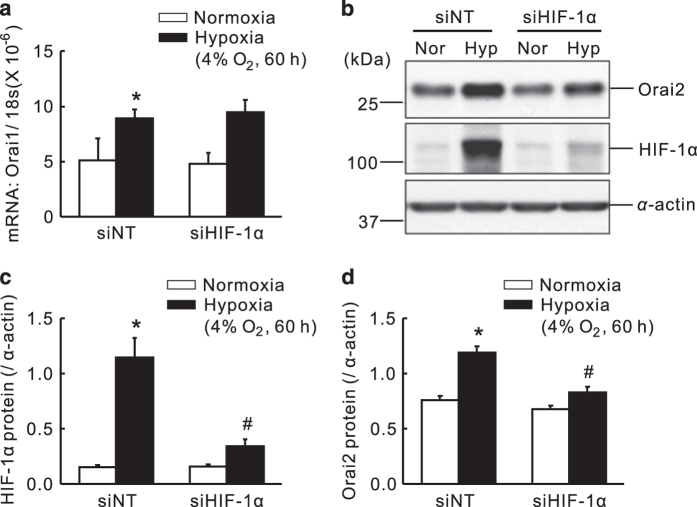
Knockdown of HIF-1*α* using siRNA transfection abolished the chronic hypoxia upregulated Orai2 expression, while not affecting the upregulation of Orai1 in rat distal PASMCs. Six hours after transfected with non-targeting (siNT) or siRNA targeting HIF-1*α* (siHIF-1*α*), PASMCs were treated with prolonged hypoxia (4% O_2_, 60 h). (**a**) Expression of Orai1 mRNA relative to 18 s mRNA measured by qRT-PCR. (**b**) Western blot showing expression of Orai2 and HIF-1*α* proteins in PASMCs. (**c** and **d**) Mean ratios of HIF-1*α* and Orai2 proteins relative to *α*-actin protein measured by western blotting in PASMCs. Bar values are means±S.E.M. (*n*=5 in each group). *P<0.05 *versus* normoxic control. ^#^*P*<0.05 *versus* hypoxic siNT control. Brackets indicate ±S.E.

Besides the HIF-1*α* transgenic mice, we also used specific small interference RNA (siRNA) against HIF-1*α* (siHIF-1*α*) to evaluate the role of HIF-1*α* in hypoxic upregulation of Orai1 and Orai2 in cultured PASMCs. First, the expression of HIF-1*α* protein was decreased by 71.7±7.7% from hypoxic exposed PASMCs treated with siHIF-1*α*, compared with that of the hypoxic non-targeted siRNA (siNT) control, indicating effective knockdown. Then, in hypoxic PASMCs, knockdown of HIF-1*α* largely abolished the hypoxic upregulation of Orai2, while not affecting the hypoxic upregulation of Orai1 (Figure 3). In combination, these results demonstrated that the hypoxic upregulation of Orai2, but not Orai1, is HIF-1*α*-dependent.

### Knockdown of Orai1 significantly reversed the hypoxic elevation of basal [Ca^2+^]_i_ and SOCE

**Figure 4:**
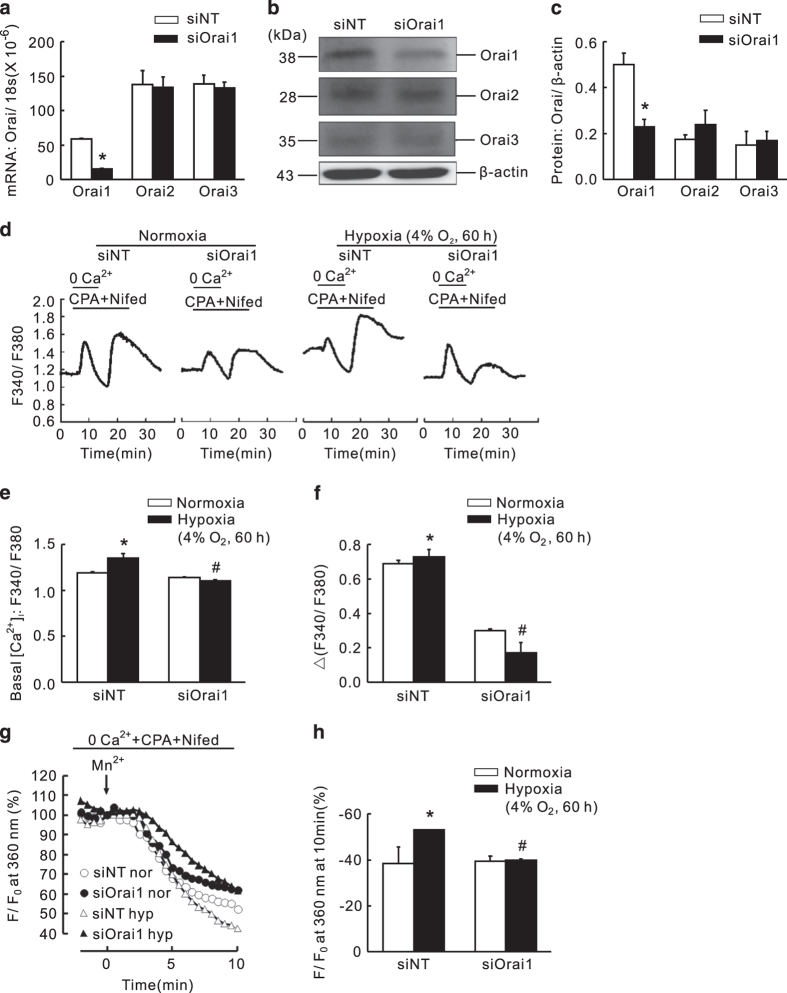
Knockdown of Orai1 inhibited chronic hypoxia-induced increases of basal [Ca^2+^]_i_ and SOCE in rat distal PASMCs. (**a**) Expression of Orai1, 2, 3 mRNA relative to 18 s mRNA measured by qRT-PCR in non-targeted small interfering RNA (siNT)- or siRNA targeted to Orai1 (siOrai1)-treated PASMCs. *P<0.05 *versus* siNT. (**b**) Western blot showing expression of Orai1, 2, 3 and *β*-actin protein in PASMCs treated with siNT or siOrai1. (**c**) Mean ratios of Orai1, 2 and 3 proteins relative to *β*-actin protein measured by western blotting in siNT- and siOrai1-treated PASMCs. *P<0.05 *versus* siNT. (**d**) Representative traces of different treatments on the time course of F340/F380 before and after restoration of extracellular Ca^2+^ in distal PASMCs perfused with Ca^2+^-free KRB solution containing 10 *μ*M CPA, 0.5 mM EGTA and 5 *μ*M nifedipine. (**e**) The effect of Orai1 silencing on hypoxia-induced changes of basal [Ca^2+^]_i_ in PASMCs. Basal [Ca^2+^]_i_ is determined as the average F340/F380 level during 0–5 min perfusion. *P<0.05 *versus* normoxic control. ^#^*P*<0.05 *versus* hypoxic siNT control. (**f**) The effect of Orai1 silencing on hypoxia-induced changes of SOCE in PASMCs. SOCE is determined as the peak increase of F340/F380 level following calcium restoration between 15 and 30 min perfusion. Bar values are means±S.E.M. (*n*=5 experiments in 74–116 cells). *P<0.05 *versus* normoxic control. ^#^*P*<0.05 *versus* hypoxic siNT control. (**g**) SOCE is determined by measuring the time course of fura-2 fluorescence intensity excited at 360 nm before and after adding 200 *μ*M Mn^2+^ in Ca^2+^-free KRB solution containing 10 *μ*M CPA and 5 *μ*M nifedipine in PASMCs. Data at each time point were normalized to fluorescence at time 0 (F/F0). (**h**) Average quenching of fura-2 fluorescence by Mn^2+^. Data are expressed as the percentage decrease in fluorescence at time 10 min from time 0. Bar values are means±S.E.M. (*n*=5 experiments in 88–109 cells). *P<0.05 *versus* normoxic control. ^#^*P*<0.05 *versus* Hypoxic siNT control. Brackets indicate ±S.E.

PASMCs were transfected with either siNT or Orai1-specific siRNA (siOrai1) and then subjected to exposure of prolonged hypoxia (4% O_2_, 60 h). Compared with that of the siNT control, the knockdown efficiency of Orai1 was 76.1±2.2% and 54.2±2.9% at mRNA and protein levels, respectively (Figures 4a–c). Meanwhile, the expression of Orai2 or Orai3 was not affected by Orai1 knockdown (Figures 4a–c), suggesting specific knockdown. First, compared with that of the normoxic control, prolonged hypoxia induced a significant increase in both basal [Ca^2+^]_i_ and SOCE (Figures 4d–h). Then, knockdown of Orai1 significantly attenuated the hypoxia-enhanced basal [Ca^2+^]_i_ by 17.7±4.2% (Figure 4e), and the hypoxia-elevated SOCE, reflected by both calcium restoration and Mn^2+^ quenching. On one hand, calcium restoration experiment revealed that compared with the normoxic control, prolonged hypoxia induced a 11.8±3.6% increase in PASMCs. Knockdown of Orai1 significantly attenuated hypoxia-induced SOCE by 77.1±4.4% (Figures 4d and f). Interestingly, treatment of siOrai1 could also decrease SOCE by 60.9±2.5% in normoxic PASMCs. On the other hand, the Mn^2+^ quenching experiment represented similar results. Prolonged hypoxia increased SOCE to 56.5±0.9% compared to that of 33.7±12.3% in normoxic PASMCs, while knockdown of Orai1 significantly decreased the hypoxia-enhanced SOCE to 39.7±1.3% (Figures 4g and h). Different with the calcium restoration experiment, knockdown of Orai1 did not affect the rate of quenching in normoxic PASMCs. The rate of quenching was 34.1±3.6% in normoxic PASMCs treated with siOrai1 *versus* 33.7±12.3% in normoxic control (Figure 4h).

### Knockdown of STIM1 significantly reversed the hypoxic elevation of basal [Ca^2+^]_i_ and SOCE

**Figure 5:**
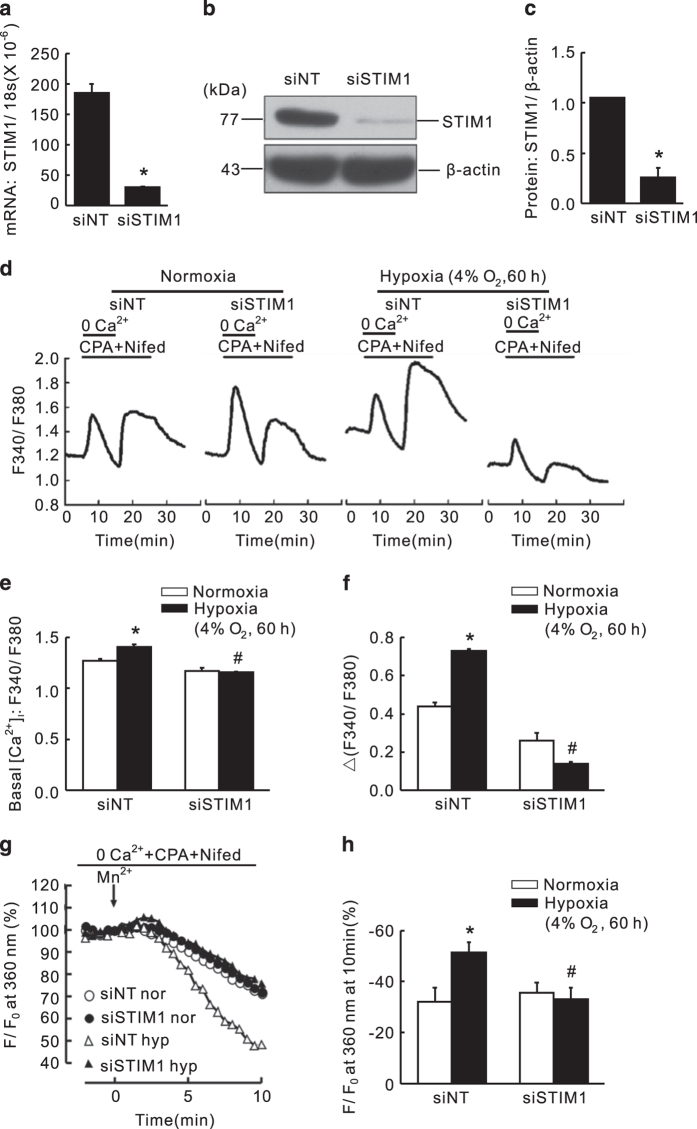
Knockdown of STIM1 inhibited chronic hypoxia-induced increases of basal [Ca^2+^]_i_ and SOCE in rat distal PASMCs. (**a**) expression of STIM1 mRNA relative to 18 s mRNA measured by qRT-PCR in non-targeted small interfering RNA (siNT)- or siRNA targeted to STIM1 (siSTIM1)-treated PASMCs. *P<0.05 *versus* siNT. (**b**) Western blot showing expression of STIM1 and *β*-actin protein in PASMCs treated with siNT or siSTIM1. (**c**) mean ratios of STIM1 protein relative to *β*-actin protein measured by Western blotting in siNT- and siSTIM1-treated PASMCs. *P<0.05 *versus* siNT. (**d**) Representative traces of different treatments on the time course of F340/F380 before and after restoration of extracellular Ca^2+^ in distal PASMCs perfused with Ca^2+^-free KRB solution containing 10 *μ*M CPA, 0.5 mM EGTA and 5 *μ*M nifedipine. (**e**) The effect of STIM1 silencing on hypoxia-induced changes of basal [Ca^2+^]_i_ in PASMCs. Basal [Ca^2+^]_i_ is determined as the average F340/F380 level during 0–5 min perfusion. *P<0.05 *versus* normoxic control. ^#^*P*<0.05 *versus* hypoxic siNT control. (**f**) The effect of STIM1 silencing on hypoxia-induced changes of SOCE in PASMCs. SOCE is determined as the peak increase of F340/F380 level following calcium restoration between 15 and 30 min perfusion. Bar values are means±S.E.M. (*n*=5 experiments in 87–133 cells). *P<0.05 *versus* normoxic control. ^#^*P*<0.05 *versus* hypoxic siNT control. (**g**) SOCE is determined by measuring the time course of fura-2 fluorescence intensity excited at 360 nm before and after adding 200 *μ*M Mn^2+^ in Ca^2+^-free KRB solution containing 10 *μ*M CPA and 5 *μ*M nifedipine in PASMCs. Data at each time point were normalized to fluorescence at time 0 (F/F0). (**h**) Average quenching of fura-2 fluorescence by Mn^2+^. Data are expressed as the percentage decrease in fluorescence at time 10 min from time 0. Bar values are means±S.E.M. (*n*=5 experiments in 90–117 cells). *P<0.05 *versus* normoxic control. ^#^*P*<0.05 *versus* hypoxic siNT control. Brackets indicate ±S.E.

In addition to Orai1, we also determined the role of STIM1 in the dysregulated intracellular calcium homeostasis in hypoxic PASMCs. Cells were transfected with either siNT or STIM1-specific siRNA (siSTIM1) and then subjected to exposure of prolonged hypoxia (4% O_2_, 60 h). Compared to that of the siNT control, the knockdown efficiency of STIM1 was 83.5±2.1% and 77.4±16.4% at mRNA and protein levels, respectively (Figures 5a–c). First, compared with that of the normoxic control, prolonged hypoxia induced a significant increase in both basal [Ca^2+^]_i_ and SOCE (Figures 5d–h). Then, knockdown of STIM1 significantly attenuated the hypoxia-enhanced basal [Ca^2+^]_i_ by 15.2±5.8% (Figure 5e), and the hypoxia-elevated SOCE, reflected by both calcium restoration and Mn^2+^ quenching. On the one hand, calcium restoration experiment revealed that compared to the normoxic control, prolonged hypoxia induced a 64.1±9.5% increase in PASMCs. Knockdown of STIM1 significantly attenuated the hypoxia-enhanced SOCE by 78.5±2.9% (Figures 5d and f). Similar to Orai1, treatment of siSTIM1 could also decrease SOCE by 37.3±9.5% in normoxic PASMCs. On the other hand, the Mn^2+^ quenching experiment represented similar results. Prolonged hypoxia increased SOCE to 50.9±6.9%, compared to that of 32.0±9.3% in normoxic PASMCs, while knockdown of Orai1 significantly decreased the hypoxia-enhanced SOCE to 32.0±9.3% (Figures 5g and h). In addition, knockdown of STIM1 did not affect the rate of quenching in normoxic PASMCs (Figure 5h).

### Knockdown of either Orai2 or Orai3 markedly inhibited the hypoxic elevation of basal [Ca^2+^]_i_ and SOCE

**Figure 6:**
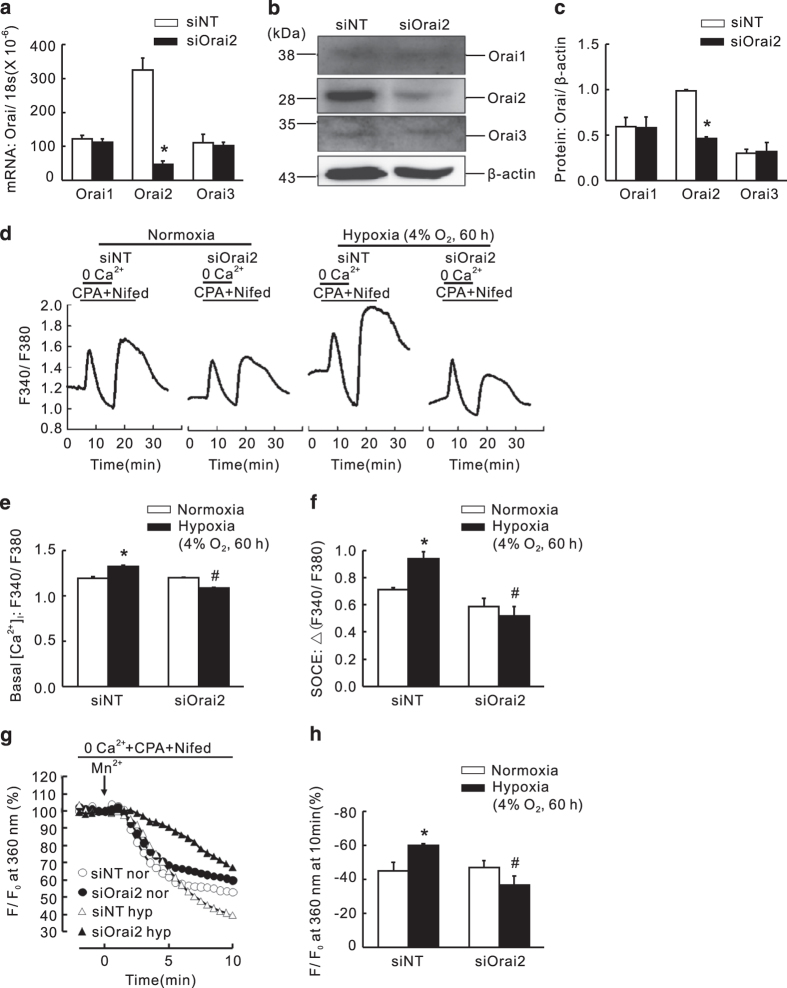
Knockdown of Orai2 inhibited chronic hypoxia-induced increases of basal [Ca^2+^]_i_ and SOCE in rat distal PASMCs. (**a**) Expression of Orai1, 2 and 3 mRNA relative to 18 s mRNA measured by qRT-PCR in non-targeted small interfering RNA (siNT)- or siRNA targeted to Orai2 (siOrai2)-treated PASMCs. *P<0.05 *versus* siNT. (**b**) Western blot showing expression of Orai1, 2, 3 and *β*-actin proteins in PASMCs treated with siNT or siOrai2. (**c**) Mean ratios of Orai1, 2 and 3 proteins relative to *β*-actin protein measured by western blotting in siNT- and siOrai2-treated PASMCs. *P<0.05 *versus* siNT. (**d**) Representative traces of different treatments on the time course of F340/F380 before and after restoration of extracellular Ca^2+^ in distal PASMCs perfused with Ca^2+^-free KRB solution containing 10 *μ*M CPA, 0.5 mM EGTA and 5 *μ*M nifedipine. (**e**) The effect of Orai2 silencing on hypoxia-induced changes of basal [Ca^2+^]_i_ in PASMCs. Basal [Ca^2+^]_i_ is determined as the average F340/F380 level during 0–5 min perfusion. *P<0.05 *versus* normoxic control. ^#^*P*<0.05 *versus* hypoxic siNT control. (**f**) The effect of Orai2 silencing on hypoxia-induced changes of SOCE in PASMCs. SOCE is determined as the peak increase of F340/F380 level following calcium restoration between 15 and 30 min perfusion. Bar values are means±S.E.M. (*n*=5 experiments in 102–115 cells). *P<0.05 *versus* normoxic control. ^#^*P*<0.05 *versus* hypoxic siNT control. (**g**) SOCE is determined by measuring the time course of fura-2 fluorescence intensity excited at 360 nm before and after adding 200 *μ*M Mn^2+^ in Ca^2+^-free KRB solution (0 Ca^2+^) containing 10 *μ*M CPA and 5 *μ*M nifedipine in PASMCs. Data at each time point were normalized to fluorescence at time 0 (F/F0). (**h**) Average quenching of fura-2 fluorescence by Mn^2+^. Data are expressed as the percentage decrease in fluorescence at time 10 min from time 0. Bar values are means±S.E.M. (*n*=5 experiments in 79–132 cells). *P<0.05 *versus* normoxic control. ^#^*P*<0.05 *versus* hypoxic siNT control. Brackets indicate ±S.E.

**Figure 7:**
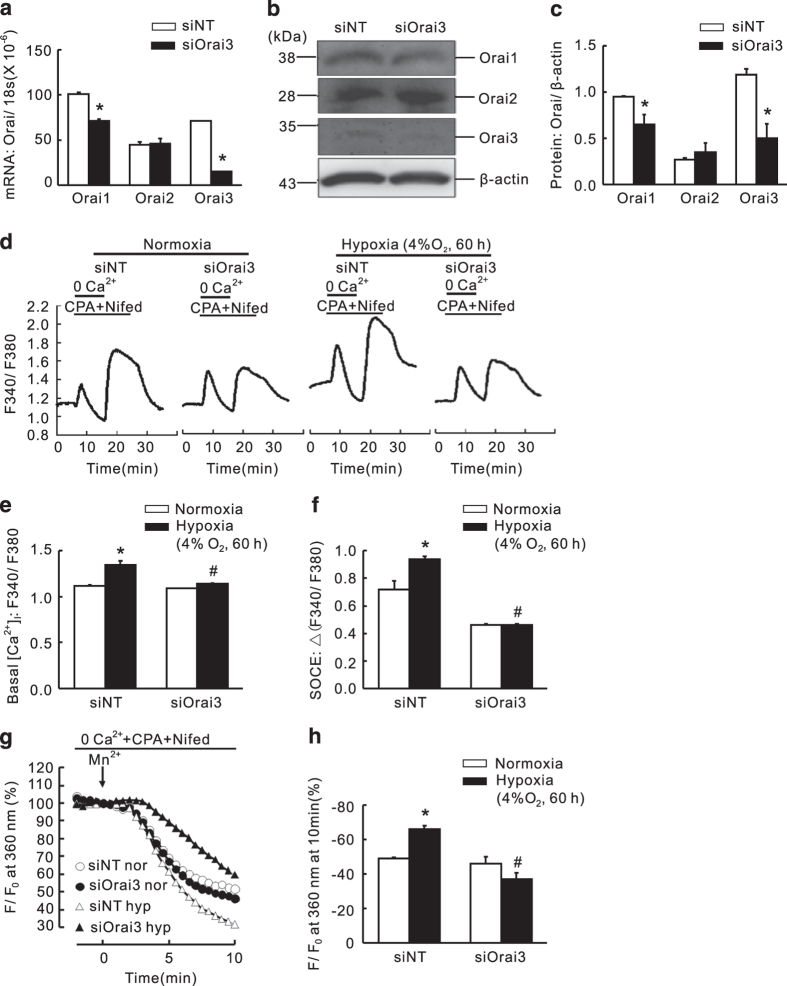
Knockdown of Orai3 inhibited chronic hypoxia-induced increases of basal [Ca^2+^]_i_ and SOCE in rat distal PASMCs. (**a**) Expression of Orai1, 2 and 3 mRNA relative to 18 s mRNA measured by qRT-PCR in non-targeted small interfering RNA (siNT)- or siRNA targeted to Orai3 (siOrai3)-treated PASMCs. *P<0.05 *versus* siNT. (**b**) Western blot showing expression of Orai1, 2, 3 and *β*-actin proteins in PASMCs treated with siNT or siOrai3. (**c**) Mean ratios of Orai1, 2 and 3 proteins relative to *β*-actin protein measured by western blotting in siNT- and siOrai3-treated PASMCs. *P<0.05 *versus* siNT. (**d**) Representative traces of different treatments on the time course of F340/F380 before and after restoration of extracellular Ca^2+^ in distal PASMCs perfused with Ca^2+^-free KRB solution containing 10 *μ*M CPA, 0.5 mM EGTA and 5 *μ*M nifedipine. (**e**) The effect of Orai3 silencing on hypoxia-induced changes of basal [Ca^2+^]_i_ in PASMCs. Basal [Ca^2+^]_i_ is determined as the average F340/F380 level during 0–5 min perfusion. *P<0.05 *versus* normoxic control. ^#^*P*<0.05 *versus* hypoxic siNT control. (**f**) The effect of Orai3 silencing on hypoxia-induced changes of SOCE in PASMCs. SOCE is determined as the peak increase of F340/F380 level following calcium restoration between 15 and 30 min perfusion. Bar values are means±S.E.M. (*n*=5 experiments in 98–121 cells). *P<0.05 *versus* normoxic control. ^#^*P*<0.05 *versus* hypoxic siNT control. (**g**) SOCE is determined by measuring the time course of fura-2 fluorescence intensity excited at 360 nm before and after adding 200 *μ*M Mn^2+^ in Ca^2+^-free KRB solution containing 10 *μ*M CPA and 5 *μ*M nifedipine in PASMCs. Data at each time point were normalized to fluorescence at time 0 (F/F0). (**h**) Average quenching of fura-2 fluorescence by Mn^2+^. Data are expressed as the percentage decrease in fluorescence at time 10 min from time 0. Bar values are means±S.E.M. (*n*=5 experiments in 81–115 cells). *P<0.05 *versus* normoxic control. ^#^*P*<0.05 *versus* hypoxic siNT control. Brackets indicate ±S.E.

Because both Orai1 and STIM1 largely contributed to SOCE in PASMCs, we then evaluated whether the other two Orai homologs, Orai2 and Orai3, also have important roles during the regulation of SOCE in PASMCs. Therefore, cultured PASMCs were transfected with either siNT, Orai2-specific siRNA (siOrai2) or Orai3-specific siRNA (siOrai3), and then subjected to exposure of prolonged hypoxia (4% O_2_, 60 h). Compared with that of the siNT control, the knockdown efficiency of was 86.7±3.8% (mRNA) and 54.5±1.2% (protein) for Orai2, and 81.0±1.4% (mRNA) and 58.6±10.0% (protein) for Orai3 (Figures 6a–c and 7a–c). First, compared with that of the normoxic control, prolonged hypoxia induced a significant increase in both basal [Ca^2+^]_i_ and SOCE (Figures 6d–h and 7d–h). Then, in parallel with that happened in knockdown of Orai1, knockdown of Orai2 or Orai3 also significantly attenuated the hypoxia-enhanced basal [Ca^2+^]_i_ (Figures 6e and 7e), and the hypoxia-elevated SOCE, reflected by both calcium restoration and Mn^2+^ quenching (Figures 6f–h and 7f–h). These results suggested that all the three Orai homologs can contribute to the regulation of SOCE in PASMCs.

## Discussion

As is well known, during the PH development, the hypoxic elevation of [Ca^2+^]_i_ due to enhanced SOCE has a key element in promoting the pulmonary vasoconstriction and proliferation, together acting as primary vessel pathology feature underlying the pathogenesis of PH. According to our previous studies, we have proved the hypoxic upregulation of either the SOCC core components (such as TRPC1 and TRPC6)^41^ or the important SOCC-regulated proteins (such as caveolin-1),^42^ all contributed to the hypoxic-triggered SOCE in PASMCs. However, whether the other SOCE-related proteins STIM1 and Orais also contribute to this process remains unknown. Therefore, in this study, by using comprehensive knockdown of STIM1 or Orais, our results suggested that knockdown of either Orai1, Orai2, Orai3 or STIM1 could significantly reverse prolonged hypoxia-induced increases of SOCE and basal [Ca^2+^]_i_ in cultured rat distal PASMCs.

Consistent with the previous studies, we discovered that hypoxic exposure significantly upregulated the expression of HIF-1*α* protein both in PAs and PASMCs. Moreover, hypoxia induced a significant upregulation of Orai1 and Orai2 at both mRNA and protein levels, but not Orai3. To determine whether the hypoxic-upregulated Orai1 and Orai2 depend on HIF-1*α*, we included both the *HIF-1*α*^+/−^* transgenic mice as *in vivo* model and specific HIF-1*α* siRNA knockdown in cultured PASMCs as *in vitro* model. Our data showed that loss of HIF-1*α* by using either heterozygous HIF-1*α* mice or siRNA knockdown markedly abolished the hypoxic upregulation of Orai2, but not Orai1, suggesting only the hypoxic upregulation of Orai2 is HIF-1*α*-dependent, whereas the hypoxic upregulation of Orai1 is dependent on other mechanism. We further determine whether Orai2 has a role in the regulation of SOCE. After knockdown Orai2, we found that downregulation of Orai2 significantly attenuated the hypoxia-increased SOCE and basal [Ca^2+^]_i_ in cultured rat distal PASMCs. These results suggest that the hypoxic upregulation of Orai2 contributes to the elevation of SOCE and basal [Ca^2+^]_i_ via stabilizing the expression of HIF-1*α*, whereas the hypoxia-elevated pathway of Orai1 expression is independent of HIF-1*α*. In view of the key role of HIF-1*α* in the development of CHPH, the Orai2 may be considered a potential target retarding pulmonary vasoconstriction and proliferation. In addition, much work have been done in evaluating the role of Orai1 in the regulation of SOCE. Similar to Orai2, downregulation of Orai1 reversed the increase of SOCE and basal [Ca^2+^]_i_ caused by CH, suggesting hypoxia-elevated Orai1 enhance SOCE and basal [Ca^2+^]_i_. But the transcriptional upregulation mechanisms for Orai1 were not evaluated clearly, what we do is at a beginning of this pathway so that further study need to be developed next. Unlike Orai1 and Orai2, we did not observe an upregulation of Orai3 expression upon hypoxic exposure, while knockdown of Orai3 could also significantly reversed the hypoxic elevation of SOCE and basal [Ca^2+^]_i_ in rat distal PASMCs, suggesting the basal level of Orai3 is also essential for the elevation of SOCE and basal [Ca^2+^]_i_. Notably, we also observed that after knockdown of Orai3, the expression of Orai1 was decreased by ~30% (Figures 7a–c). As we know, Orai3 was reported to be an important component of ARC entry.^43^ Shuttleworth *et al.*^44^ reported that STIM1 is required for ARC channel activation and that both Orai1 and Orai3 contribute subunits to ARC channels. Using various preassembled concatenated Orai1–Orai3 multimers, Shuttleworth group further reported that the molecular architecture of ARC channels is a pentameric assembly of three Orai1 and two Orai3 subunits. Moreover, Charlotte Dubois *et al.*^45^ demonstrated *in vitro* models that enhanced Orai3 expression favors heteromerization with Orai1 to form a novel channel to support store-independent Ca2^+^ entry. Thus, one possible explanation is that as Orai3 could not form SOCCs by its own, knockdown of Orai3 might cause a proportion of Orai1 that could not combine with Orai3 to form CRAC or ARC channels, thus entered a pathway of protein degradation. Therefore, the loss of hypoxia-enhanced SOCE could be explained by the loss of Orai1. Regarding this point, additional studies need to be conducted in the near future to uncover the detail mechanisms about whether Orai3 has a direct impact on the decrease of basal [Ca^2+^]_i_ and SOCE.

In the previous study, Ng *et al.*^46,47^ found that Orai1 and STIM1, as well as TRPC1, can form molecular complex to mediate SOCE in mouse PASMCs, suggesting a central role of STIM1 in SOCE. Hou *et al.*^48^ found that CH upregulated the expression of STIM1 both on mRNA and protein levels in rat distal PA. That is contradictory to the findings of our present study. In the present study, we found that whether at mRNA level or at protein level, CH failed to alter the expression of STIM1. Hou *et al.*^48^ found that knockdown of STIM1 using STIM1-specific siRNA reversed the enhancement of SOCE in rat PASMCs exposed to prolonged hypoxia. This finding is consistent with the results of our present study. But the PASMCs used by Hou *et al.* were passaged for 3–8 times, while our cells were primary cultured. We believe that the characteristics of PASMCs may vary through the process. Moreover, we used Mn^2+^ quenching method to assess SOCE, which is also different of the study conducted by Hou *et al.* As is known to all, Mn^2+^ quenching is the gold standard of SOCE assessment, for it can eliminate the effect of ER/SR releasing Ca^2+^ to the cytoplasma, thus reflecting the Ca^2+^ influx from the extracellular. In our present study, we found that knockdown of STIM1 reversed CH-increased basal [Ca^2+^]_i_ in rat distal PASMCs, and the effect of STIM1 silencing on CH-enhanced SOCE was verified by Mn^2+^ quenching method. Together, we found that knockdown of STIM1 attenuated SOCE in hypoxic PASMCs, suggesting a triggering role of STIM1 in initialing SOCE upon intracellular calcium store depletion.

**Figure 8:**
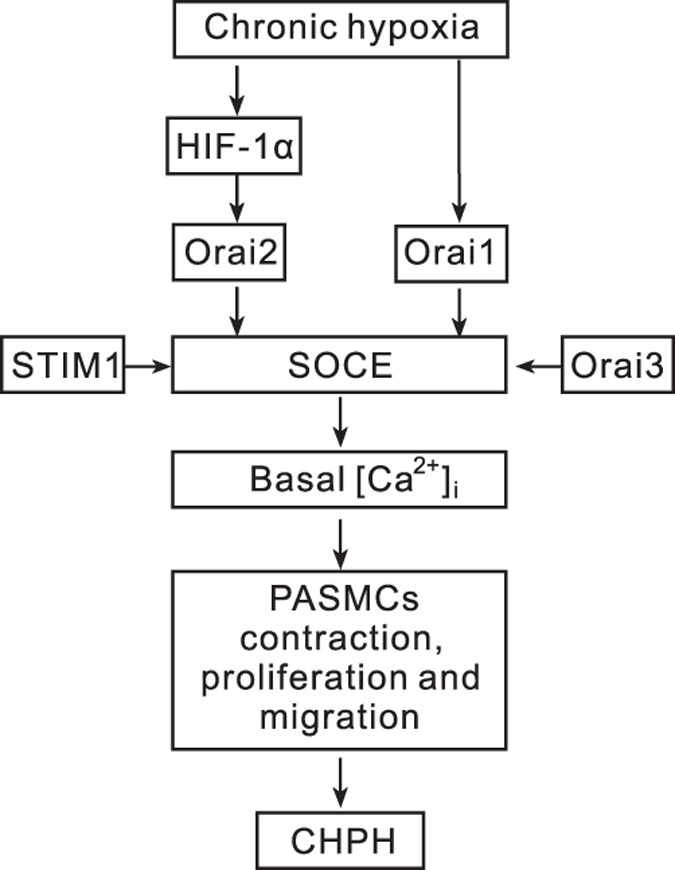
Schematic representation of the hypothesized regulation–signaling axis of HIF-1*α*/Orai/STIM1 mechanism.

An outline was shown in Figure 8. In summary, in this study, we found the following: (1) chronic hypoxic exposure stabilized the expression of HIF-1*α*, leading to upregulation of Orai2 and a subsequent enhancement of SOCE and basal [Ca^2+^]_i_, whereas hypoxia upregulates the expression of Orai1 in a HIF-1*α*-independent manner; and (2) knockdown of either STIM1 or Orai family proteins (Orai1, Orai2 and Orai3) could attenuate the hypoxia-elevated SOCE and basal [Ca^2+^]_i_ in PASMCs. As elevation of basal [Ca^2+^]_i_ leads to contraction, proliferation and migration of PASMCs, eventually the pathogenesis of CHPH, blockage of the elevation of basal [Ca^2+^]_i_ would reverse the process and prevent the genesis of CHPH. Our results added knowledge that the Orais and STIM1 could act as novel therapeutic targets for the treatment against CHPH.

## Materials and methods

### Animals

Animal protocols were approved by the Animal Care and Use Committee of Guangzhou Medical University. Sprague–Dawley rats (male, 175–300 g) were purchased from Guangdong Medical Laboratory Animal Center (Guangzhou, China). HIF-1*α* heterozygous-mutant (*HIF-1α*^*+/–*^) mice were genotyped as described before.^49^ All mice were housed in specific pathogen-free facilities. Littermate mice from *HIF-1α*^*+/+*^×*HIF-1α*^*+/–*^ mating were genotyped at 3–4 weeks old.

### Exposure of animals to chronic hypoxia

Rats (male, 175–300 g) or mice (male, 8 weeks old) were placed in a hypoxia chamber for 21 days to establish the chronic hypoxia-induced PH animal model, as previously described.^50^ The chamber was continuously flushed with a mixture of room air and N_2_ to maintain 10±0.5% O_2_ and CO_2_<0.5%. The chamber O_2_ concentration was continuously monitored using a PRO-OX unit (RCI Hudson, Anaheim, CA, USA). Animals were exposed to room air for 10 min twice a week for changing cage and replenishing food and water. Normoxic control animals were kept in room air next to the hypoxic chamber.

### Isolation of distal PA and culture of PASMCs

The distal intrapulmonary arteries (⩾fourth generation) were dissected from normoxic or chronic hypoxic rats and mice, as we previously described.^51^ PASMCs were enzymatically isolated and plated onto 25 mm glass coverslips (Fisher Scientific, Pittsburgh, PA, USA).^52^ Rat PASMCs were cultured in Dulbecco’s modified Eagle’s medium (DMEM) containing 1 g/l D-glucose (Life Technologies, Carlsbad, CA, USA) with 10% fetal bovine serum (Life Technologies) and 1% penicillin–streptomycin (MP Biomedicals, Solon, OH, USA) until 60–70% confluence prior each experiment.

### siRNA transfection and prolonged hypoxic exposure of PASMCs

siRNAs targeting to rat HIF-1*α* (siHIF-1*α*), Orai1 (siOrai1), Orai2 (siOrai2), Orai3 (siOrai3), STIM1 (siSTIM1) and siNT used as negative control were designed and synthesized by GenePharma (Suzhou, China). Rat PASMCs were transfected with 50 nM siHIF-1*α*, siOrai1, siOrai3, siSTIM1, siNT or 100 nM siOrai2 using HiPerFect Transfection Reagent (Qiagen,Valencia, CA, USA) at 6 *μ*l/ml as transfection vehicle in 0 serum DMEM. After 6 h transfection, serum was added to a final concentration of 0.3% and PASMCs were exposed to normoxia or hypoxia (4% O_2_) for 60 h. Knockdown efficiency was evaluated by quantitative real-time PCR (qRT-PCR) and western blotting.

### Measurement of intracellular Ca^2+^ concentration

After incubation with 7.5 *μ*M fura-2 (Invitrogen, Carlsbad, CA, USA) for 60 min at 37 °C under an atmosphere of 5% CO_2_–95% air, coverslips with PASMCs were mounted in a closed polycarbonate chamber clamped in a heated aluminum platform (PH-2; Warner Instrument, Hamden, CT, USA) on the stage of a Nikon TSE 100 Ellipse inverted microscope (Melville, NY, USA). The chamber was perfused at 1 ml/min with Krebs–Ringer bicarbonate (KRB) solution, which contained (in mM) 118 NaCl, 4.7 KCl, 0.57 MgSO_4_, 1.18 KH_2_PO_4_, 25 NaHCO_3_, 2.5 CaCl_2_ and 10 glucose. To assess SOCE, we perfused PASMCs for at least 10 min with Ca^2+^-free KRB containing 5 *μ*M nifedipine to prevent calcium entry through L-type VDCCs and 10 *μ*M CPA to deplete SR calcium stores. KRB perfusate also contained 0.5 mM EGTA to chelate any residual Ca^2+^. SOCE was assessed in two ways. First, we measured [Ca^2+^]_i_ at 12 s intervals before and after restoration of extracellular [Ca^2+^] to 2.5 mM. SOCE was evaluated from the increase in [Ca^2+^]_i_ caused by restoration of extracellular [Ca^2+^] in the continued presence of cyclopiazonic acid (CPA) and nifedipine. Second, we monitored fura-2 fluorescence excited at 360 nm at 30 s intervals before and after addition of MnCl_2_ (200 *μ*M) to the cell perfusate. SOCE was evaluated from the rate at which fura-2 fluorescence was quenched by Mn^2+^, which enters the cell as a Ca^2+^ surrogate and reduces fura-2 fluorescence on binding to the dye. Fluorescence excited at 360 nm is the same for Ca^2+^-bound and Ca^2+^-free fura-2; therefore, changes in fluorescence can be assumed to be caused by Mn^2+^ alone. Quenching was quantified as the change in F360 (ΔF360) measured from 5 to 15 min and expressed as a percentage of F360 at 5 min.

### Real-time quantitative PCR

Total RNA in de-endothelialized distal PA and PASMCs was extracted using TRIzol method.^34^ Reverse transcription was performed using PrimeScript RT Reagent Kit (Takara, Japan). The reaction mixture contained 1 *μ*g total RNA in a 20 *μ*l volume. cDNA was quantified by qRT-PCR using QuantiTect SYBR Green PCR Master Mix (Qiagen) in a iCycler IQ real-time PCR detection system (BioRad, Hercules, CA, USA) using the following conditions: 95 °C for 15 min and 45 cycles, each at 94 °C for 15 s, 57.5 °C for 20 s and 72 °C for 20 s. The volume of each qRT-PCR reaction mixture was 25 *μ*l containing 300 nM forward and reverse primers and cDNA template from 50 ng RNA. Primer sequences of rat Orai1, Orai2, Orai3, STIM1 and 18 s were designed using Primer3 software (http://simgene.com/Primer3), and are shown as follows, where S is sense and AS is antisense:

Orai1-S (5′- GATGAGCCTCAACGAGCACT-3′),

Orai1-AS (5′- GACTTCCACCATCGCTACCA-3′),

Orai2-S (5′- GTGGGTCTCATCTTCGTGGT-3′),

Orai2-AS (5′- CCACCTGTAGGCTTCTCTCG-3′),

Orai3-S (5′- GCCCAGCTTTAGACTGTTGC-3′),

Orai3-AS (5′- CTGAGCAGGAATTTGGCTTC-3′),

STIM1-S (5′- ATGCCAATGGTGATGTGGAT-3′),

STIM1-AS (5′- CCATGGAAGGTGCTGTGTTT-3′),

18s-S (5′- GCAATTATTCCCCATGAACG-3′),

18s-AS (5′- GGCCTCACTAAACCATCCAA-3′).

Identity of the qPCR products was confirmed by (1) a single sharp peak in the melting curve performed after cDNA amplification, (2) a single band of the expected size resolved by agarose gel electrophoresis and (3) DNA sequencing. Melting curves were performed at 95 °C for 1 min and 55 °C for 1 min, followed by 80 increments of 0.5 °C at 10 s intervals. qRT-PCR detection threshold cycle (C_T_) values were generated by iCycler IQ software. Relative concentration of each transcript was calculated using the Pfaffl method.^53^ Efficiency for each gene was determined from five-point serial dilutions of positive control cDNA samples.

### Western blotting

Total proteins in tissues or cells were extracted in ice-cold RIPA buffer (Biocolors, Shanghai, China) containing 1% NP40, 0.1% SDS, 5 mM EDTA, 0.5% sodium deoxycholate and 1% 100 mM PMSF. De-endothelialized distal PA was homogenized using ultrasonication. PASMCs were washed with phosphate buffered saline and homogenized by scraping in lysis buffer on ice and then were span down to remove cell debris. Total protein concentration in homogenate samples were determined by BCA protein assay (Pierce, Rockford, IL, USA). Samples were combined with 5× loading buffer, boiled and resolved on SDS-PAGE calibrated with prestained protein molecular weight markers. Separated proteins were transferred to polyvinylidene difluoride membranes (pore size 0.2 *μ*M; Millipore, Billerica, MA, USA). After blocking with 5% non-fat dry milk in TBS-0.1% Tween-20, membranes were blotted with mouse monoclonal antibody for HIF-1*α* (Clone H1a67; Novus Biologicals Inc., Littleton, CO, USA), *α*-actin (Sigma-Aldrich), *β*-actin (Sigma-Aldrich, St Louis, MO, USA), rabbit polyclonal antibodies for Orai1, Orai2 (Santa Cruz Biotechnology, Santa Cruz, CA, USA) and Orai3 (Prosci, Poway, CA, USA), or rabbit monoclonal antibodies for STIM1 (Abcam, Cambridge, UK). The membranes were then washed and probed with anti-rabbit or anti-mouse horseradish peroxidase-conjugated IgG (KPL, Inc., Gaithersburg, MD, USA). The bound antibodies were detected using an enhanced luminol-linked chemiluminescence detection system (ECL, GE Healthcare, Piscataway, NJ, USA).

### Reagents and drugs

Unless otherwise specified, all reagents were obtained from Sigma-Aldrich. Stock solutions (30 mM) of CPA and nifedipine were made in pluronic dimethyl sulfoxide (DMSO, Invitrogen). Fura-2 AM (Invitrogen) was prepared on the day of the experiment as a 2.5 mM stock solution in DMSO.

### Statistical analysis

Statistical analyses were conducted using Student’s *t*-test for two groups and one-way ANOVA for multiple groups of data. Differences were considered significant when *P*< 0.05. Data are presented as means±S.E.M.; ‘*n*’ refers to the sample size (that is, the number of the animals providing PAs or primary culture of PASMCs).

## Additional information

**Publisher’s note:** Springer Nature remains neutral with regard to jurisdictional claims in published maps and institutional affiliations.

## References

[bib1] Simonneau G, Gatzoulis MA, Adatia I, Celermajer D, Denton C, Ghofrani A et al. Updated clinical classification of pulmonary hypertension. J Am Coll Cardiol 2013; 62(25 Suppl): D34–D41. 24355639 10.1016/j.jacc.2013.10.029

[bib2] Golovina VA, Platoshyn O, Bailey CL, Wang J, Limsuwan A, Sweeney M et al. Upregulated TRP and enhanced capacitative Ca(2+) entry in human pulmonary artery myocytes during proliferation. Am J Physiol Heart Circ Physiol 2001; 280: H746–H755. 11158974 10.1152/ajpheart.2001.280.2.H746

[bib3] Brough GH, Wu S, Cioffi D, Moore TM, Li M, Dean N et al. Contribution of endogenously expressed Trp1 to a Ca2+-selective, store-operated Ca2+ entry pathway. FASEB J 2001; 15: 1727–1738. 11481220

[bib4] Jiang Q, Lu W, Yang K, Hadadi C, Fu X, Chen Y et al. Sodium tanshinone IIA sulfonate inhibits hypoxia-induced enhancement of SOCE in pulmonary arterial smooth muscle cells via the PKG-PPAR-gamma signaling axis. Am J Physiol Cell Physiol 2016; 311: C136–C149. 27194472 10.1152/ajpcell.00252.2015 PMC4967135

[bib5] Smyth JT, Dehaven WI, Jones BF, Mercer JC, Trebak M, Vazquez G et al. Emerging perspectives in store-operated Ca2+ entry: roles of Orai, Stim and TRP. Biochim Biophys Acta 2006; 1763: 1147–1160. 17034882 10.1016/j.bbamcr.2006.08.050

[bib6] Liao Y, Erxleben C, Yildirim E, Abramowitz J, Armstrong DL, Birnbaumer L. Orai proteins interact with TRPC channels and confer responsiveness to store depletion. Proc Natl Acad Sci USA 2007; 104: 4682–4687. 17360584 10.1073/pnas.0611692104 PMC1838661

[bib7] Peel SE, Liu B, Hall IP. ORAI and store-operated calcium influx in human airway smooth muscle cells. Am J Respir Cell Mol Biol 2008; 38: 744–749. 18239188 10.1165/rcmb.2007-0395OC PMC2643203

[bib8] Lai N, Lu W, Wang J. Ca(2+) and ion channels in hypoxia-mediated pulmonary hypertension. Int J Clin Exp Pathol 2015; 8: 1081–1092. 25972995 PMC4396234

[bib9] Varnai P, Hunyady L, Balla T. STIM and Orai: the long-awaited constituents of store-operated calcium entry. Trends Pharmacol Sci 2009; 30: 118–128. 19187978 10.1016/j.tips.2008.11.005 PMC3125588

[bib10] Berra-Romani R, Mazzocco-Spezzia A, Pulina MV, Golovina VA. Ca2+ handling is altered when arterial myocytes progress from a contractile to a proliferative phenotype in culture. Am J Physiol Cell Physiol 2008; 295: C779–C790. 18596214 10.1152/ajpcell.00173.2008 PMC2544451

[bib11] Demuro A, Penna A, Safrina O, Yeromin AV, Amcheslavsky A, Cahalan MD et al. Subunit stoichiometry of human Orai1 and Orai3 channels in closed and open states. Proc Natl Acad Sci USA 2011; 108: 17832–17837. 21987805 10.1073/pnas.1114814108 PMC3203769

[bib12] Derler I, Madl J, Schutz G, Romanin C. Structure, regulation and biophysics of I(CRAC), STIM/Orai1. Adv Exp Med Biol 2012; 740: 383–410. 22453951 10.1007/978-94-007-2888-2_16

[bib13] Lee KP, Yuan JP, Hong JH, So I, Worley PF, Muallem S. An endoplasmic reticulum/plasma membrane junction: STIM1/Orai1/TRPCs. FEBS Lett 2010; 584: 2022–2027. 19944100 10.1016/j.febslet.2009.11.078 PMC2866752

[bib14] Liao Y, Erxleben C, Abramowitz J, Flockerzi V, Zhu MX, Armstrong DL et al. Functional interactions among Orai1, TRPCs, and STIM1 suggest a STIM-regulated heteromeric Orai/TRPC model for SOCE/Icrac channels. Proc Natl Acad Sci USA 2008; 105: 2895–2900. 18287061 10.1073/pnas.0712288105 PMC2268556

[bib15] Madl J, Weghuber J, Fritsch R, Derler I, Fahrner M, Frischauf I et al. Resting state Orai1 diffuses as homotetramer in the plasma membrane of live mammalian cells. J Biol Chem 2010; 285: 41135–41142. 20961852 10.1074/jbc.M110.177881 PMC3003411

[bib16] Soboloff J, Rothberg BS, Madesh M, Gill DL. STIM proteins: dynamic calcium signal transducers. Nat Rev Mol Cell Biol 2012; 13: 549–565. 22914293 10.1038/nrm3414 PMC3458427

[bib17] Mercer JC, Dehaven WI, Smyth JT, Wedel B, Boyles RR, Bird GS et al. Large store-operated calcium selective currents due to co-expression of Orai1 or Orai2 with the intracellular calcium sensor, Stim1. J Biol Chem 2006; 281: 24979–24990. 16807233 10.1074/jbc.M604589200 PMC1633822

[bib18] DeHaven WI, Smyth JT, Boyles RR, Putney JJ. Calcium inhibition and calcium potentiation of Orai1, Orai2, and Orai3 calcium release-activated calcium channels. J Biol Chem 2007; 282: 17548–17556. 17452328 10.1074/jbc.M611374200

[bib19] Lis A, Peinelt C, Beck A, Parvez S, Monteilh-Zoller M, Fleig A et al. CRACM1, CRACM2, and CRACM3 are store-operated Ca2+ channels with distinct functional properties. Curr Biol 2007; 17: 794–800. 17442569 10.1016/j.cub.2007.03.065 PMC5663639

[bib20] Hoth M, Penner R. Depletion of intracellular calcium stores activates a calcium current in mast cells. Nature 1992; 355: 353–356. 1309940 10.1038/355353a0

[bib21] Hoth M, Penner R. Calcium release-activated calcium current in rat mast cells. J Physiol 1993; 465: 359–386. 8229840 10.1113/jphysiol.1993.sp019681 PMC1175434

[bib22] Cai X. Molecular evolution and structural analysis of the Ca(2+) release-activated Ca(2+) channel subunit, Orai. J Mol Biol 2007; 368: 1284–1291. 17400243 10.1016/j.jmb.2007.03.022

[bib23] Gwack Y, Srikanth S, Feske S, Cruz-Guilloty F, Oh-hora M, Neems DS et al. Biochemical and functional characterization of Orai proteins. J Biol Chem 2007; 282: 16232–16243. 17293345 10.1074/jbc.M609630200

[bib24] Hou X, Pedi L, Diver MM, Long SB. Crystal structure of the calcium release-activated calcium channel Orai. Science 2012; 338: 1308–1313. 23180775 10.1126/science.1228757 PMC3695727

[bib25] Mignen O, Thompson JL, Shuttleworth TJ. Both Orai1 and Orai3 are essential components of the arachidonate-regulated Ca2+-selective (ARC) channels. J Physiol 2008; 586: 185–195. 17991693 10.1113/jphysiol.2007.146258 PMC2375546

[bib26] Liou J, Kim ML, Heo WD, Jones JT, Myers JW, Ferrell JJ et al. STIM is a Ca2+ sensor essential for Ca2+-store-depletion-triggered Ca2+ influx. Curr Biol 2005; 15: 1235–1241. 16005298 10.1016/j.cub.2005.05.055 PMC3186072

[bib27] Manji SS, Parker NJ, Williams RT, van Stekelenburg L, Pearson RB, Dziadek M et al. STIM1: a novel phosphoprotein located at the cell surface. Biochim Biophys Acta 2000; 1481: 147–155. 11004585 10.1016/s0167-4838(00)00105-9

[bib28] Roos J, DiGregorio PJ, Yeromin AV, Ohlsen K, Lioudyno M, Zhang S et al. STIM1, an essential and conserved component of store-operated Ca2+ channel function. J Cell Biol 2005; 169: 435–445. 15866891 10.1083/jcb.200502019 PMC2171946

[bib29] Oh-hora M, Rao A. Calcium signaling in lymphocytes. Curr Opin Immunol 2008; 20: 250–258. 18515054 10.1016/j.coi.2008.04.004 PMC2574011

[bib30] Feske S, Gwack Y, Prakriya M, Srikanth S, Puppel SH, Tanasa B et al. A mutation in Orai1 causes immune deficiency by abrogating CRAC channel function. Nature 2006; 441: 179–185. 16582901 10.1038/nature04702

[bib31] Wu MM, Buchanan J, Luik RM, Lewis RS. Ca2+ store depletion causes STIM1 to accumulate in ER regions closely associated with the plasma membrane. J Cell Biol 2006; 174: 803–813. 16966422 10.1083/jcb.200604014 PMC2064335

[bib32] Wang Y, Deng X, Hewavitharana T, Soboloff J, Gill DL. Stim, ORAI and TRPC channels in the control of calcium entry signals in smooth muscle. Clin Exp Pharmacol Physiol 2008; 35: 1127–1133. 18782202 10.1111/j.1440-1681.2008.05018.x PMC3601895

[bib33] Putney JJ. Capacitative calcium entry: sensing the calcium stores. J Cell Biol 2005; 169: 381–382. 15866892 10.1083/jcb.200503161 PMC2171919

[bib34] Lu W, Wang J, Peng G, Shimoda LA, Sylvester JT. Knockdown of stromal interaction molecule 1 attenuates store-operated Ca2+ entry and Ca2+ responses to acute hypoxia in pulmonary arterial smooth muscle. Am J Physiol Lung Cell Mol Physiol 2009; 297: L17–L25. 19395668 10.1152/ajplung.00063.2009 PMC2711802

[bib35] Shuttleworth TJ. STIM and Orai proteins and the non-capacitative ARC channels. Front Biosci (Landmark Ed) 2012; 17: 847–860. 22201777 10.2741/3960 PMC3295580

[bib36] Thompson JL, Shuttleworth TJ. A plasma membrane-targeted cytosolic domain of STIM1 selectively activates ARC channels, an arachidonate-regulated store-independent Orai channel. Channels (Austin) 2012; 6: 370–378. 22992514 10.4161/chan.21947 PMC3508776

[bib37] Hawkins BJ, Irrinki KM, Mallilankaraman K, Lien YC, Wang Y, Bhanumathy CD et al. S-glutathionylation activates STIM1 and alters mitochondrial homeostasis. J Cell Biol 2010; 190: 391–405. 20679432 10.1083/jcb.201004152 PMC2922639

[bib38] Xiao B, Coste B, Mathur J, Patapoutian A. Temperature-dependent STIM1 activation induces Ca(2)+ influx and modulates gene expression. Nat Chem Biol 2011; 7: 351–358. 21499266 10.1038/nchembio.558 PMC3097298

[bib39] Ritchie MF, Samakai E, Soboloff J. STIM1 is required for attenuation of PMCA-mediated Ca2+ clearance during T-cell activation. EMBO J 2012; 31: 1123–1133. 22246182 10.1038/emboj.2011.495 PMC3298001

[bib40] Ottolia M, John S, Xie Y, Ren X, Philipson KD. Shedding light on the Na+/Ca2+ exchanger. Ann N Y Acad Sci 2007; 1099: 78–85. 17446447 10.1196/annals.1387.044

[bib41] Wang J, Weigand L, Lu W, Sylvester JT, Semenza GL, Shimoda LA. Hypoxia inducible factor 1 mediates hypoxia-induced TRPC expression and elevated intracellular Ca2+ in pulmonary arterial smooth muscle cells. Circ Res 2006; 98: 1528–1537. 16709899 10.1161/01.RES.0000227551.68124.98

[bib42] Yang K, Lu W, Jiang Q, Yun X, Zhao M, Jiang H et al. Peroxisome proliferator-activated receptor gamma-mediated inhibition on hypoxia-triggered store-operated calcium entry. A caveolin-1-dependent mechanism. Am J Respir Cell Mol Biol 2015; 53: 882–892. 26020612 10.1165/rcmb.2015-0002OC PMC4742941

[bib43] Gonzalez-Cobos JC, Zhang X, Zhang W, Ruhle B, Motiani RK, Schindl R et al. Store-independent Orai1/3 channels activated by intracrine leukotriene C4: role in neointimal hyperplasia. Circ Res 2013; 112: 1013–1025. 23349245 10.1161/CIRCRESAHA.111.300220 PMC3658112

[bib44] Shuttleworth TJ. Arachidonic acid, ARC channels, and Orai proteins. Cell Calcium 2009; 45: 602–610. 19278724 10.1016/j.ceca.2009.02.001 PMC2744097

[bib45] Dubois C, Vanden AF, Lehen'Kyi V, Gkika D, Guarmit B, Lepage G et al. Remodeling of channel-forming ORAI proteins determines an oncogenic switch in prostate cancer. Cancer Cell 2014; 26: 19–32. 24954132 10.1016/j.ccr.2014.04.025

[bib46] Ng LC, Ramduny D, Airey JA, Singer CA, Keller PS, Shen XM et al. Orai1 interacts with STIM1 and mediates capacitative Ca2+ entry in mouse pulmonary arterial smooth muscle cells. Am J Physiol Cell Physiol 2010; 299: C1079–C1090. 20739625 10.1152/ajpcell.00548.2009 PMC2980318

[bib47] Ng LC, McCormack MD, Airey JA, Singer CA, Keller PS, Shen XM et al. TRPC1 and STIM1 mediate capacitative Ca2+ entry in mouse pulmonary arterial smooth muscle cells. J Physiol 2009; 587(Pt 11): 2429–2442. 19332490 10.1113/jphysiol.2009.172254 PMC2714011

[bib48] Hou X, Chen J, Luo Y, Liu F, Xu G, Gao Y. Silencing of STIM1 attenuates hypoxia-induced PASMCs proliferation via inhibition of the SOC/Ca2+/NFAT pathway. Respir Res 2013; 14: 2. 23289723 10.1186/1465-9921-14-2 PMC3599439

[bib49] Iyer NV, Kotch LE, Agani F, Leung SW, Laughner E, Wenger RH et al. Cellular and developmental control of O2 homeostasis by hypoxia-inducible factor 1 alpha. Genes Dev 1998; 12: 149–162. 9436976 10.1101/gad.12.2.149 PMC316445

[bib50] Yu AY, Shimoda LA, Iyer NV, Huso DL, Sun X, McWilliams R et al. Impaired physiological responses to chronic hypoxia in mice partially deficient for hypoxia-inducible factor 1alpha. J Clin Invest 1999; 103: 691–696. 10074486 10.1172/JCI5912 PMC408131

[bib51] Lu W, Wang J, Shimoda LA, Sylvester JT. Differences in STIM1 and TRPC expression in proximal and distal pulmonary arterial smooth muscle are associated with differences in Ca2+ responses to hypoxia. Am J Physiol Lung Cell Mol Physiol 2008; 295: L104–L113. 18424621 10.1152/ajplung.00058.2008 PMC2494791

[bib52] Wang J, Shimoda LA, Sylvester JT. Capacitative calcium entry and TRPC channel proteins are expressed in rat distal pulmonary arterial smooth muscle. Am J Physiol Lung Cell Mol Physiol 2004; 286: L848–L858. 14672922 10.1152/ajplung.00319.2003

[bib53] Pfaffl MW. A new mathematical model for relative quantification in real-time RT-PCR. Nucleic Acids Res 2001; 29: e45. 11328886 10.1093/nar/29.9.e45 PMC55695

